# Differences of embedding adipose-derived stromal cells in natural and synthetic scaffolds for dermal and subcutaneous delivery

**DOI:** 10.1186/s13287-020-02132-5

**Published:** 2021-01-19

**Authors:** Frederik Penzien Mamsen, Lea Munthe-Fog, Mikela Karen Mungal Kring, Dominik Duscher, Mikkel Taudorf, Adam J. Katz, Stig-Frederik Trojahn Kølle

**Affiliations:** 1 Department of Plastic Surgery, Stemform, Gyngemose Parkvej 74, DK-2860 Copenhagen, Denmark; 2 Department of Plastic and Hand Surgery, Klinikum rechts der Isar, Technical University of Munich, Ismaningerstrasse 22, 81675 Munich, Germany; 3 Department of Radiology, Rigshospitalet, University Hospital of Copenhagen, Blegdamsvej 9, DK-2100 Copenhagen, Denmark; 4 Department of Plastic and Reconstructive Surgery, Wake Forest University School of Medicine, Medical Center Boulevard, Winston-Salem, NC USA

**Keywords:** Adipose-derived stromal cells, Scaffolds, Viability, Angiogenesis, Volume retention, Cell delivery

## Abstract

**Background:**

In recent years, adipose-derived stromal cells (ASCs) have been heavily studied for soft tissue regeneration, augmentation, and dermal wound healing.

**Methods:**

In this review, we investigated the trends in injectable scaffolds for ASC delivery in the dermis, and injectable or implantable scaffolds for ASC delivery in the subcutis. A total of 547 articles were screened across three databases; of these, 22 studies were found to be eligible and were included. The scaffolds were subdivided and analyzed based on their tissue placement (dermis or subcutis), delivery method (injected or implanted), and by the origin of the materials (natural, synthetic, and combinatory).

**Results:**

ASCs embedded in scaffolds generally showed improved viability. Neovascularization in the transplanted tissue was greater when undifferentiated ASCs were embedded in a combinatory scaffold or if differentiated ASCs were embedded in a natural scaffold. ASCs embedded in natural materials underwent more adipogenic differentiation than ASCs embedded in synthetic scaffolds, indicating an etiologically unknown difference that has yet to be described. Increased mechanical strength of the scaffold material correlated with improved outcome measurements in the investigated studies*.* Wound healing studies reported reduced healing time in all except one article due to contraction of the control wounds.

**Conclusions:**

In future clinical trials, we recommend embedding ASCs in injectable and implantable scaffolds for enhanced protection, retained viability, and improved therapeutic effects.

**Trial registration:**

This review was registered with PROSPERO: ID=CRD42020171534.

**Graphical abstract:**

The use of scaffolds as a vehicle for ASC delivery generally improved cell viability, angiogenesis, and wound healing in vivo compared to utilizing ASCs alone. ASCs embedded in natural materials induced more adipogenesis than ASCs embedded in synthetic materials. Adipogenic-induced ASCs further increased this effect. The included studies indicate that the seeded scaffold material influences the differentiation of ASCs in vivo*.* All studies investigating the mechanical strength of ASC scaffolds reported improved outcome measurements with improved mechanical strength. The results suggest that scaffolds, in general, are favorable for ASC delivery. We recommend initiating clinical studies using scaffolds based on mechanical properties and tunability to improve ASC viability. For fat regeneration, natural scaffolds are recommended. 
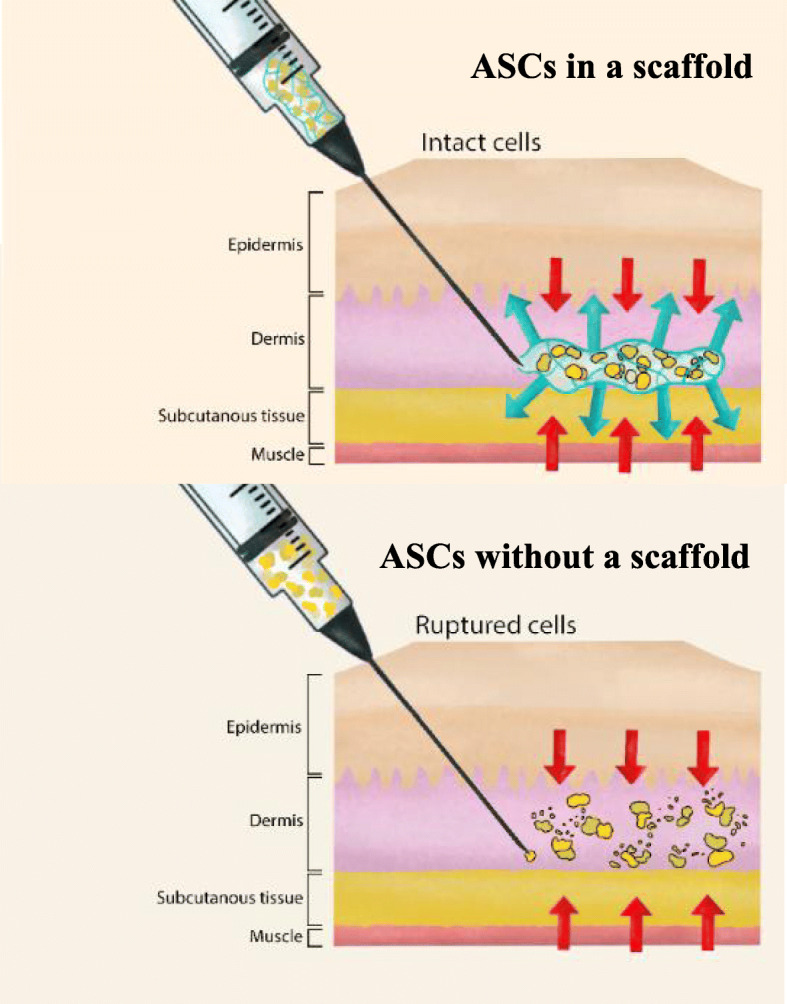

## Background

Mesenchymal stem cells (MSCs) are under investigation in hundreds of clinical trials [1]. Adipose-derived stromal/stem cells (ASCs) are currently one of the most utilized MSCs in plastic and reconstructive surgery because procurement is minimally invasive (e.g., liposuction), and ASCs are abundant in number and readily obtained from fat. Soft tissue regeneration and dermal wound healing compose a fraction of the studies investigating ASC-based therapy. The rationale is their immunomodulatory and regenerative qualities [2]. However, there should be a greater focus on methods for cell product delivery in clinical trials, as cell quality and potency are highly important for a successful clinical outcome. ASCs are commonly suspended in low-viscosity liquid proven to damage cells during injection, thus reducing the cell count and effect of treatment [3, 4]. Culturing ASCs for treatment is currently extremely costly and time-consuming. It is therefore essential to maximize cell vitality and minimize cell damage and rupture during injection.

Soft tissue defects following surgery, birth defects, or traumas are currently restored using major flap surgery or artificial prostheses, e.g., breast prostheses. Fat grafting is an alternative method for volume restoration. However, retention rates can be low and unpredictable [5–7] and often require multiple procedures for esthetically pleasing results. Specialists have explored ways to improve fat graft retention. At present, ASC enrichment of fat grafts has shown the most promising results in two human randomized controlled clinical trials [8, 9]. Their secretion of angiogenic and anti-apoptotic factors [10] can theoretically increase fat-tissue depend neo-angiogenesis after transplantation and lead to an improved graft retention. Although encouraging clinical results have been reported, the reproducibility, reliability, and improved procedure of the soft tissue restoration remain to be investigated.

The application of ASCs in dermal wound healing has been well investigated. Every surgical specialty has patients with healing problems and hypertrophic scarring, and the application of ASCs has been acknowledged as an improved treatment in this area. ASCs are being investigated due to their ability to mediate and modulate immune responses associated with wound healing. Wounds are normally dependent on the healthy surrounding extracellular matrix (ECM), cells, and serum for vascularization and remodeling of the wound bed [11]. ASC is known to secrete a plethora of regenerative growth factors [12] that improve the quality and extent of wound healing.

To improve clinical results, scaffolds as vehicles for ASCs are being explored for their protective properties. Scaffolds are known to influence graft quality, tissue formation, and wound healing by maintaining ASC viability/retention and metabolic activity and improving neoangiogenesis and thereby graft retention and wound healing.

In this study, we searched for literature (in accordance with PICO guidelines) to review the use of scaffolds to improve the ASC delivery for subcutaneous and dermal applications.

### Background for cell delivery scaffolds

Scaffolds are supportive materials. They are currently extensively investigated for tissue engineering and drug delivery. Fundamentally, a scaffold must define a 3D architectural space that can provide structural support for the transplanted cells and integrate easily with the surrounding tissue [13]. Scaffolds are especially of interest for injectable cell delivery, as they theoretically protect the cells during injection, resulting in more viable cells with retained potency, thus increasing the rate of successful clinical treatments with minimal scaring [14].

Designing a scaffold requires careful consideration of multiple aspects that all may affect the quality of the graft and ultimately the success of the treatment. As such, porosity, degradation, mechanical properties, signaling molecules, and polymer type play a role in the quality of hydrogel scaffolds [13]. The structural base of scaffolds should ideally mimic the natural 3D environmental niche of the recipient tissue for an easy transition from culture to tissue [15]. When choosing a scaffold, it is important to understand the native abilities of the scaffolded material and the nature of the recipient site. Injectable scaffolds may be favorable in settings such as drug delivery or for minimal scaring in cosmetic and reconstructive surgery. Implantable scaffolds may however be favorable in an environmental niche where the mechanical strength is of most importance such as for meniscus repair. Here, we investigate whether embedding ASCs in scaffolds can improve the quality of subcutaneous soft tissue augmentation and dermal wound healing in comparison to solely injecting/implanting ASCs. We further inspect whether the origin of the scaffold material (natural, synthetic, or combinatory) impacts the ASCs, vascularization, differentiation, and quality of the grafts.

### Natural and synthetic scaffolds

Natural scaffolds inherently play a role in biological signaling, cell adhesion, and can easily be degraded and remodeled in vivo. However, they lack mechanical strength and controllable degradation. Natural scaffolds can be subject to batch-to-batch variation, thus variate in quality and quantity [16]. Synthetic scaffolds often have tunable mechanical properties, controllable degradation, and are without batch-to-batch variance. Theoretically, synthetic polymers will however not facilitate cell signaling or cell differentiation [16]. By combining natural and synthetic scaffolds the desired scaffold qualities could offer a scaffold with biological signaling, cell adherence, controlled degradation, batch consistency, tunability, and mechanical strength.

Another property of scaffolds is their ability to support ASCs towards adipocytes. The bio-mechanism is however vastly unclear. A factor known is however the stiffness of the ECM. A stiffer ECM generally facilitates osteogenesis and a softer ECM adipogenesis. This change is due to the changes in focal adhesions. ASCs will change their morphology by spreading wider in a stiffer matrix and regulate focal adhesions leading to osteogenesis via beta-catenin signaling and adipogenesis via PPAR-gamma in a softer matrix [17].

## Methods

The protocol was published in the register of PROSPERO and approved on March 17, 2020, with registration number CRD42020171534.

### Search

Studies from MEDLINE, Embase, and Cochrane Library were identified. Titles and abstracts were screened for eligibility using the inclusion criteria until November 1, 2019. The search was limited to the English language. The search strings were modified for use in the different databases.

### Inclusion criteria

We included peer reviewed publications assessing transplantation of ASCs embedded in injectable scaffolds into the dermis layer in vivo or injectable and implantable scaffolds into the subcutaneous layer in vivo. Studies on the topical application of ASCs, e.g., by acellular dermal matrix for burn wounds, were excluded. Furthermore, studies employing animals subjected to chronic illnesses such as diabetes were excluded. To ensure a degree of confidence, all the included studies compared their results to a control group, e.g., transplantation of cells without a scaffold. Eligible studies also included an assessment of ASC viability post ejection or transplantation, graft retention, or vascularization in comparison to a control group.

Study characteristics were assessed using the PRISMA PICOS guidelines.

### Outcomes

The eligible principal results were viability, potency, ASC and graft retention, vascularization, adipogenesis, and wound healing.

This included viability both in vitro and in vivo; potency was defined as the ability of ASCs to proliferate in culture and by metabolic activity, e.g., MTT assay, vascularization by immunostaining or histology, adipogenesis by oil red O or Nile red staining, dermal thickness was assessed by histology and wound healing assessed by wound closure and epithelial thickness. Furthermore, eligible retention results included ASC detection in vivo using, e.g., fluorescence and graft retention assessed by MRI or histology.

In addition, eligible studies should include an assessment of scaffold biocompatibility and biodegradability in vitro using, e.g., a gravimetric method, or an assessment of retention in vivo using, e.g., histology or volume retention.

## Results

### Characteristics of included studies

Xenogeneic models (human/murine) were the preferred model in most of the studies (*n* = 17), whereas only six studies investigated a true murine model whereof one was in an autologous setting. In the nonxenogeneic models, all animals used in the individual studies were of the same species and breed. No human clinical trials were found. An outline of the included studies is depicted in Table 1. None of the articles described randomized housing of the animals or blinding of the caregivers.

**Table 1:** Outline of the included studies1

Cell graft	Subcutanous injection	Dermal injection	Subcutaneous implant
Autologe	*N* = 1 [18]	*N* = 0	*N* = 0
Allogenic	*N* = 2 [19, 20]	*N* = 2 [21, 22]	*N* = 1 [23]
Xenogenic	*N* = 7 [14, 24–29]	*N* = 3 [30–32]	*N* = 6 [33–38]

Confirmation of the cell lineage (ASC) should be performed before application of ASCs, either by trilineage differentiation or flow cytometry for cell surface markers. Six of the 22 included articles confirmed ASC surface markers using flow cytometry prior to application [20–22, 25, 29, 37], of which two studies included trilineage differentiation [21, 22]. In all the included studies, the ASCs were culture expanded prior to application, and in 12 studies, ASCs were cultured for more than 24 h in their respective scaffolds prior to in vivo application [21, 24–26, 28, 29, 32–34, 36–38].

Following scaffold loading either by mixing the scaffold with the cells or by culturing the cells in the scaffold, the scaffolds were either injected subcutaneously, intradermally, or implanted under the skin of the animal subjects. Subcutaneous injections were assessed in ten studies using 18–28G needles with cell concentrations ranging from 0.05–17 × 10^6^ ASC/ml (Tables 2 and 3), five articles studied dermal injections using 23–25G needles with cell concentrations ranging from 0.01–2 × 10^6^ ASC/ml (Tables 4 and 5), and seven articles studied the subcutaneous implantation of ASCs (Tables 6 and 7).

**Table 2:** Characteristics of subcutaneous injections of ASCs embedded in scaffolds2

Author (year)	Type of scaffold	Subtype (intervention)	Specimens	ASCs	Gauge	Placement	ASCs/ml	Number
**Cai et al. (2015)** [14]	Copolymer	^a^PEG with 1 wt% ^b^PNIPAM (SHIELD-1) + ASCs	Athymic nude mice	Human	28 G	Dorsum	16,665,000	5
		^a^PEG with 0.7 wt% ^b^PNIPAM (SHIELD-0.7) + ASCs						
		^a^PEG with w/o ^b^PNIPAM (SHIELD-0) + ASCs						
**Linh et al. (2017)** [24]		^c^CHPA-^d^GTA-^e^PDGF-BB + ASCs	BALB/c nude mice	Human	NR	Dorsum	10,000,000	4
		^c^CHPA-^d^GTA + ASCs						
**Chen et al. (2017)** [18]	Copolymer + protein	Laminin-alginate beads + ASCs	Sprague-Dawley rats	Rats Autologous	NR	Dorsum	50,000	3
		Adipocytes + laminin-alginate beads + ASCs						
**Choi et al. (2006)** [25]	Polymer	^f^PLGA + dASCs	Athymic nude mice	Human	23 G	Neck	1,000,000	6
**Choi et al. (2009)** [26]	ECM	Human ECM powder + ASCs	BALB/c mice	Human	18 G	Dorsum	1,000,000	10
**Kim et al. (2012)** [27]	Polysaccharide	Alginate + dASCs	Nude mice	Human	23 G	Chest wall and abdomen	~ 1,000,000	5
**Cheung et al. (2013)** [28]	^g^DAT + (GAG + polymer) or (polysaccharide + polymer)	^h^MGC + 5% ^g^DAT + dASCs	Wistar rats	Human	NR	NR	~ 1,000,000	3
		^i^MCS + 5% ^g^DAT + dASCs						
**Wang et al. (2013)** [29]	ECM	^j^SISE + ASCs	Nude mice	Human	NR	Dorsum	100,000	6
		^k^ATE + ASCs						
**Sumi et al. (2013)** [19]	Protein + anticoagulant	^l^F/P, plasma, ^m^FGF-2 and ASCs	Fischer 344 rats	Rats	25 G	Dorsum	4,000,000	NR
		^l^F/P and plasma and ASCs						
		Plasma and ^m^FGF-2 and ASCs						
		^l^F/P, serum and ^m^FGF-2 and ASCs						
		^l^F/P and serum and ASCs						
		Serum and ^m^FGF-2 and ASCs						
**Derby et al. (2014)** [20]	Peptide	^n^PuraMatrix + ASCs	Nude mice	Murine	23 G	Parascapular	5,000,000	6

*dASCs* adipogenically induced ASCs, *NR* not reported, ^a^*PEG* polyethylene glycol, ^b^*PNIPAM* poly(N-isopropylacrylamide), ^c^*CHPA* 4-hydroxyphenyl acetic acid, ^d^*GTA* gelatin modified with tyramine, ^e^*PDGF-BB* platelet-derived growth factor, ^f^*PLGA* poly (lactide-co-glycolide), ^g^*DAT* decellularized adipose tissue, ^h^*MGC* methacrylated glycol chitosan, ^i^*MCS* methacrylated chondroitin sulfate, ^j^*SISE* small intestine submucosa extract, ^k^*ATE* adipose tissue extract, ^l^*F/P* fragmin/protamine, ^m^*FGF-2* fibroblast growth factor 2, ^n^*PuraMatrix* PuraMatrix peptide hydrogel (BD Biosciences, Bedford, Massachusetts)

**Table 3:** Results of subcutaneous injections of ASCs embedded in scaffolds.2

Author (year) [ref]	Scaffold material	Cells	Results from each study’s best scaffold for ASC delivery		Duration in vivo
			In vitro	In vivo	
**Cai et al.** (2015) [14]	^a^PEG + ^b^PNIPAM (SHIELD-1)	ASCs	Cells are protected from shear stress during injection	Cell retention 60% at day 3 vs. 13% for ASCs alone ASC proliferation within the scaffold	2 weeks
**Linh et al.** (2017) [24]	^c^CHPA-^d^GTA-^e^PDGF-BB	ASCs	Cells are viable and proliferate in scaffold	Cells are viable and proliferate within the scaffold Observed infiltration and vascularization of the scaffold	2 weeks
**Chen et al.** (2017) [18]	Adipocytes in laminin-alginate beads	ASCs	n/a	Fat graft retention at week 6 is 60% and increasing to 89% at 12 weeks, indicating formation of new fat tissue. Newly formed tissue with healthy adipocytes	12 weeks
**Choi et al.** (2006) [25]	^f^PLGA	dASCs	Cells proliferate and maintain pre-adipogenic phenotype in the scaffold	Cells maintain their pre-adipocyte phenotype in the scaffold Newly formed tissue following week 4 Neovascularization observed	8 weeks
**Choi et al.** (2009) [26]	hECM powder	ASCs	Cells adhere and proliferate in ECM powder	Observed infiltration and vascularization of the scaffold Newly formed tissue with healthy adipocytes either by differentiation or migration of host cells	8 weeks
**Kim et al.** (2012) [27]	Alginate	dASCs	Cells maintain their pre-adipocyte phenotype in the scaffold	Fat graft retention at week 10 is 50% ± 12%, indicating that the entire scaffold had been resorbed and half of it replaced by neotissue Newly formed healthy fat tissue with vascularization	10 weeks
**Cheung et al.** (2013) [28]	^g^MCS + ^h^DAT	dASCs	Cells are viable and maintain their pre-adipocyte phenotype in the scaffold	Observed infiltration and vascularization of the scaffold Newly formed tissue with healthy adipocytes Graft surrounded by a fibrous capsule that qualitatively decreased in thickness and increased in vascularization as the scaffold remodeled.	12 weeks
**Wang et al.** (2013) [29]	^i^ATE	ASCs	Cells proliferate in the scaffold	Confirmed adipogenesis within the scaffold. Observation of vascular components in the scaffold.	8 weeks
**Sumi et al.** (2013) [19]	^j^F/P, plasma, ^k^FGF-2 (a)	ASCs	Cells proliferate in the scaffold	Observed vascularization of the scaffold at day 15	8 weeks
**Derby et al.** (2014) [20]	^l^PuraMatrix (amino acids)	ASCs	n/a	Cells are viable and proliferate within the scaffold ASC display ability to transdifferentiate into epithelial cells	8 weeks

*dASCs* adipogenically induced ASCs, ^a^*PEG* polyethylene glycol, ^b^*PNIPAM* poly(N-isopropylacrylamide), ^c^*CHPA* 4-hydroxyphenyl acetic acid, ^d^*GTA* gelatin modified with tyramine, ^e^*PDGF-BB* platelet-derived growth factor, ^f^*PLGA* poly (lactide-co-glycolide), ^g^*MCS* methacrylated chondroitin sulfate, ^h^*DAT* decellularized adipose tissue, ^i^*ATE* adipose tissue extract, ^j^*F/P* fragmin/protamine, ^k^*FGF-2* fibroblast growth factor 2, ^l^*PuraMatrix* PuraMatrix peptide hydrogel (BD Biosciences, Bedford, Massachusetts)

**Table 4:** Characteristics of dermal injections of ASCs embedded in scaffolds

Author (year)	Scaffold material	Subtype (intervention)	Specimens	ASCs	Gauge	Placement	ASCs/ml	Number
**Dong et al. (2014)** [21]	Polymer + protein	^a^PEG and ^b^SH-HA + ASCs	Sprague-Dawley Rats	Rats	NR	Dorsum	1,000,000	3
**Dong et al. (2017)** [22]	Polysaccharide & polymer	^a^PEG and gelatin + ASCs	FVB mice	Murine	NR	Dorsum	1,000,000	10
**Machula et al. (2014)** [30]	Protein	Electrospun tropoelastin + ASCs	SCID congenic mice	Human	NR	Dorsum	756,000	6
**Kim et al. (2016)** [31]	Polymer + ECM	ECM protein + methylcellulose +ASCs	Sprague-Dawley rats	Human	23 G	Dorsum	100,000	3
**Cheng et al. (2017)** [32]	Polysaccharide + protein	Chitosan + ^c^bFGF + ASCs	C57/B6 mice	Human	25 G	Dorsum	2,000,000	4
		Chitosan/gelatin+ ASCs + ^c^bFGF						

*dASCs* adipogenically differentiated ASCs, ^a^*PEG* polyethylene glycol, ^b^*SH-HA* thiolated hyaluronic acid, ^c^*bFGF* basic fibroblast growth factor

**Table 5:** Results of dermal injections of ASCs embedded in scaffolds

Author (year) [ref]	Scaffold material	Cells	Results from each study’s best scaffold for ASC delivery		Duration in vivo
			In vitro	In vivo	
**Dong et al.** (2014) [21]	^a^PEG + bSH − HA	ASCs	Cells are viable in the scaffold	Decreased wound contraction Re-epithelialization from the wound edges The scaffold retains the ASCs within the scaffold. No ASCs were found in host tissue Increased vascularization of the wounds	3, 7, and 14 days
**Dong et al.** (2017) [22]	^a^PEG + gelatin	ASCs	Cells are viable and proliferate in the scaffold The scaffolds mechanical strength decreased during 4 weeks of culture. ASCs regenerate the ECM network and maintain scaffold shape.	Cells are viable within the scaffold Faster wound healing Increased vascularization of the wounds	4 weeks
**Machula et al.** (2014) [30]	Electrospun tropoelastin	ASCs	The cells are compatible with the scaffold assessed by ASC morphology and deposition of ECM	Faster wound healing Thicker re-epithelization of wounds	6 days
**Kim et al.** (2016) [31]	ECM protein + methylcellulose	ASCs	Cells are viable and proliferate in the scaffold	Observed host infiltration of the scaffold No increased vascularization nor increased epithelial thickness of the wounds	3 weeks
**Cheng et al.** (2017) [32]	Chitosan/gelatin + ^c^bFGF	ASCs	Cells are viable and proliferate in the scaffold 1.2% of the cells are released after 14 days	Increased vascularization HNA+ Cells in the wound 11 ± 3.2% CD31+ cells per power field	5 days

*dASCs* adipogenically differentiated ASCs, ^a^*PEG* polyethylene glycol, ^b^*SH-HA* thiolated hyaluronic acid, ^c^*bFGF* basic fibroblast growth factor

**Table 6:** Characteristics of subcutaneously implanted ASCs embedded in scaffolds4

Author (year)	Type	Subtype (intervention)	Specimen	ASCs	Placement	ASC/ml	Number
**Wu et al. (2017)** [33]	Biodegradable polymer	^a^PLGA and ^b^OEG_1_ + ASCs	Nude mice	Huabman	Dorsum	500,000	14
		^a^PLGA and ^c^OEG_9_ + ASCs					
**Zhang et al. (2017)** [34]		^a^PLGA + dASCs	Nude mice	Human	Dorsum	NR	5
		^a^PLGA + ASCs					
**Cho et al. (2005)** [35]		Fibrin gel, ^d^bFGF + dASCs	Athymic nude mice	Human	Dorsum	80,000,000	4
		Fibrin gel, ^d^bFGF, ^e^PGA + ^f^PLLA + dASCs					
**Hong et al. (2006)** [36]	Gelatin	Gelatin sponge + dASCs	SCID mice	Human	Dorsum	3,000,000	4
		Gelatin sponge + ASCs					
**Dhillon et al. (2019)** [37]	Polysaccharide + peptide sequence	^g^MGC + ASCs	NOD/SCID mice	Human	Dorsum	10,000,000	6
		^g^MGC-^h^RGD + ASCs					
		^g^MGC-^i^IKVAV + ASCs					
**Jing et al. (2007)** [23]	Polysaccharide	Alginate +ASCs	BALB/c mice	Murine	Dorsum	2,000,000	8
		Alginate +dASCs					
**Storck et al. (2017)** [38]	Biodegradable polymer and protein	^j^Pu-fibrin + ASCs	Athymic mice	Human	Groin	1,000,000	5
		^j^Pu-fibrin + dASCs					
		^j^Pu-fibrin +dASCs + fat flap					

*dASCs* adipogenically differentiated ASCs, ^a^*PLGA* poly (lactide-co-glycolide), ^b^*OEG*_*1*_ ethylene glycol, ^c^*OEG*_*9*_ oligo (ethylene glycol) 400, ^d^*bFGF* basic fibroblast growth factor, ^e^*PGA* poly (glycolic acid), ^f^*PLLA* poly(L-lactic acid), ^g^*MGC* N-methacrylate glycol chitosan, ^h^*RGD* GGGGRGDS peptide sequence derived from collagen and fibronectin, ^i^*IKVAV* CSRARKQAASIKVAVSADR peptide sequence derived from laminin, ^j^*PU* poly(ε-caprolactone)-based polyurethane

**Table 7:** Results of subcutaneously implanted ASCs embedded in scaffolds

Author (year) [ref]	Scaffold material	Cells	Results from each study’s best scaffold for ASC delivery		Duration in vivo
			In vitro	In vivo	
**Wu et al.** (2017) [33]	^a^PLGA + ^b^OEG_9_	ASCs	Most cells are viable and proliferate after 3 days The cells adhere to the scaffold	Increased vascularization Host cells invade the scaffold Positive stain for CD31+ cells and VEGF	4 weeks
**Zhang et al.** (2017) [34]	^a^PLGA	dASCs	Cells are viable and adhere to the scaffold Cells form lipid droplets and express adipogenic genes	Increased vascularization Fat formation surrounded by a thin fibrotic capsule No necrosis or inflammation Positive staining for CD31+ cells and vWF	12 weeks
**Cho et al.** (2005) [35]	Fibrin gel + ^c^bFGF + ^d^PGA + ^e^PLLA	dASCs	Cells proliferate in the scaffold Cells form lipid droplets	The implant did not shrink at visual inspection Fat formation	6 weeks
**Hong et al.** (2006) [36]	Gelatin sponge	dASCs	Cells proliferate in the scaffold Cells form lipid droplets	Increased vascularization Human dASCs found after 4 weeks Fat formation (40% of histological section is newly formed fat tissue)	4 weeks
**Dhillon et al.** (2019) [37]	^f^MGC-^g^RGD	ASCs	Cells are viable and metabolically active Cells express angiogenic genes Cells have normal morphology	Cells are retained inside the scaffold Increased vascularization	2 weeks
**Jing et al.** (2007) [23]	Alginate	dASCs	Cells form lipid droplets Cells express adipogenic genes and proteins	The grafted dASCs and scaffold are visually like adipose tissue Increased metabolic activity of dASCs during the first week Fat formation and adipogenic gene expression	8 weeks
**Storck et al.** (2017) [38]	^j^Pu-fibrin + fat flap	dASCs	n/a	Fat formation Neotissue originates from the host	12 weeks

*dASCs* adipogenically differentiated ASCs, ^a^*PLGA* poly (lactide-co-glycolide), ^b^*OEG*_*9*_ oligo (ethylene glycol) 400, ^c^*bFGF* basic fibroblast growth factor, ^d^*PGA* poly (glycolic acid), ^e^*PLLA* poly(L-lactic acid), ^f^*MGC* N-methacrylate glycol chitosan, ^g^*RGD* GGGGRGDS peptide sequence derived from collagen and fibronectin, ^j^*PU* poly(ε-caprolactone)-based polyurethane

### The scaffold

A scaffold used in a subcutaneous/dermal setting should serve as a delivery vehicle for ASCs and possess a certain mechanical stability to protect the cells against shearing and pressure-associated damage during delivery. Once the ASCs are delivered, the scaffold should provide structural support for the cells to attach, proliferate, and differentiate. Furthermore, the scaffold should provide a void volume for vascularization and new tissue formation. As such, the engineered material determines the scaffold functionality, biodegradability, and compatibility [39].

In the literature, frequently assessed scaffold materials are natural polymers (polysaccharides, gelatin, extracellular matrix (ECM) components) and synthetic polymers, most frequently PLGA or PEG, or combinations of the two. In general, natural polymers have been reported to display excellent biocompatibility [12]. The cells are most often able to adhere and proliferate in a natural scaffold; however, the mechanical stability of the scaffold is limited. It is therefore often attempted to reinforce natural scaffolds with synthetic polymers, providing the physical properties necessary for successful delivery [39]. However, this reinforcement can potentially compromise scaffold biocompatibility either by evoking an immunological reaction or by altering the degradational properties of the scaffold. As such, biodegradation is yet another important aspect when designing a scaffold for cell delivery. Depending on the purpose, a scaffold should have at least a temporary resistance to biodegradation upon implantation but eventually become degraded over time (days to months) without evoking an immunological response. As the majority of the included studies established xenogeneic models using immunocompromised animals to overcome mismatched cell grafting [18, 21, 28], immunological evaluation of biocompatibility is not included in this review.

In the eligible studies, considerations regarding the scaffold design led to the utilization of one of three categories of material: natural polymers (polysaccharides, gelatin, extracellular matrix (ECM) components) (*n* = 13); synthetic polymers (*n* = 4), such as PLGA; or combinations of the two (*n* = 5).

Although the length of the studies limited the evaluation of the total degradation time, studies evaluating natural [18, 24, 28] or synthetic [14, 20] scaffolds reported at least partial degradation of scaffolds at the end of the study periods (Table 8). Based on these limited results, the natural and synthetic scaffold materials included in this study seem to meet the defined criteria for biodegradation. However, full graft retention was not extensively evaluated, as the scaffold materials had not fully degraded at the evaluation point, which could lead to misconception of the retention rates [18, 23, 27, 35].

**Table 8:** Degradation time of the scaffold materials

**Scaffold**	**Origin**	**Degradation in vivo**	**Measured after**
^a^PEG [14]	Synthetic	70%	3 days
^a^PEG /^b^PNIPAM [14]	Synthetic	40%	3 days
^c^CHPA / ^d^GTA / ^e^PDGF-BB [24]	Natural	40%	2 weeks
Laminin/alginate [18]	Natural	60%	6 weeks
^f^PuraMatrix [20]	Synthetic	100%	8 weeks
^h^MGC/ ^g^DAT [28]	Natural	50%	12 weeks
^i^MCS / ^g^DAT [28]	Natural	75–80%	12 weeks
**Scaffold**	**Origin**	**Degradation in vitro**	**Measured after**
^c^CHPA-^d^GTA-^e^PDGF-BB [24]	Natural	50%	20 days
Chitosan/gelatin + ^j^bFGF [32]	Natural	65 ± 3.5%	1 week
^k^PLGA and ^l^OEG_9_ [33]	Combinatory	70%	4 weeks
^k^PLGA [34]	Synthetic	≈50%	6 weeks

^a^*PEG* polyethylene glycol, ^b^*PNIPAM* poly(*N*-isopropylacrylamide), ^c^*CHPA* 4-hydroxyphenyl acetic acid, ^d^*GTA* gelatin modified with tyramine, ^e^*PDGF-BB* platelet-derived growth factor, ^f^*PuraMatrix* PuraMatrix peptide hydrogel (BD Biosciences, Bedford, Massachusetts), ^g^*DAT* decellularized adipose tissue, ^h^*MGC* methacrylated glycol chitosan, ^i^*MCS* methacrylated chondroitin sulfate, ^j^*bFGF* basic fibroblast growth factor, ^k^*PLGA* poly (lactide-co-glycolide), ^l^*OEG*_*9*_ oligo (ethylene glycol) 400

### Cell characteristics

When evaluating the ASCs in the included studies, some precautions should be taken. Promising results have been obtained in the field of stem cell therapy, but the differences in proprietary methods for cell culture and production have resulted in clinical results with variable success. In regard to these methods, the crucial question of cell dose remains. With respect to the variation in cell culture procedures, the cell dose needed to achieve a clinically relevant result is still vastly unknown. A recent porcine study investigated the optimal dose of ASC enrichment to fat grafts within the range of 2.5–20 × 10^6^ cells/ml. A concentration of > 10 × 10^6^ significantly increased graft retention compared with the nonenriched control (*p* = 0.02). However, no significant dose dependency in graft retention was found [40], implying that a certain threshold cell dose is needed for successful fat graft retention.

None, but one, of the 22 included articles in this review tested the effects of various cell concentrations [22]. In the included study by Dong et al. [22], a cell concentration of 10 × 10^6^ cells/ml displayed a significantly increased in vitro expression of the two stemness-related genes Oct4 and Sox2. The expression of the wound healing-related cytokine genes Sdf-1, Hgf, Angpt-1, Vegf-α, Fgf-2, and Pigf was also elevated when compared to a lower cell concentration of 1 × 10^6^ cells/ml (*p* < 0.05). This result, in context with the previous porcine study [40], indicates that higher cell concentrations may be favorable for fat tissue engineering in terms of graft retention, stemness, and wound healing.

### ASC viability and potency

Intuitively, the quality of the cells embedded into a scaffold affects the fate of the transplant. If the fraction of viable/vital cells loaded into the scaffold is low, the procedure of mixing and injecting the cells may further impair the cell quality and ultimately jeopardize the clinical results. Thus, the cell quality should be assessed prior to application and, if possible, monitored post ejection. Parameters such as cellular metabolism, proliferation, and population doubling time can provide crucial information about the quality of the cell product prior to application and thereby insinuate the treatment outcome. Twelve of the included articles assessed ASC proliferation in coculture with the scaffold [19, 21, 22, 24–26, 28, 29, 32–34, 37]. Nine articles found the ASCs to be proliferating, but without indication of a preferred scaffold material (natural (*n* = 5)) [19, 24, 26, 29, 32], combinatory (*n* = 2) [22, 33], or synthetic (*n* = 2) [25, 34]. Three articles, of which two tested the natural scaffold material methacrylated glycol chitosan [23, 29], reported restricted proliferation resulting in a maintained or decreased cell population. The third study utilized a combinatory scaffold comprised of hyaluronic acid-SH and a PEG scaffold [21].

The results confirm that it is possible for the ASCs to proliferate and maintain healthy coculture with the tested scaffold materials, both of natural and synthetic origin, barring only a few exceptions.

Cells have been shown to rupture when injected due to three different types of mechanical forces [14]: a pressure drop across the cell, shearing forces due to linear shear flow, and stretching forces due to extensional flow [4]. Six of the included studies investigated the effects of mechanical strength by, e.g., oscillatory rheology, on cell viability in vitro. In these studies, increased mechanical strength of the scaffold generated retained viability of the ejected ASCs. The retained viability was regardless of the origin of scaffold material [14, 21, 31, 33, 34, 37] (*n* = 2 natural, *n* = 2 synthetic, and *n* = 2 combinatory) when compared to ASCs injected in a low viscus solution, e.g., saline solution.

### Neo adipogenesis

A scaffold’s ability to support adipogenic differentiation is crucial, as these scaffolds are meant to regenerate and augment soft tissue. The demarcation of the origin of scaffolds in natural, synthetic, or combinatory scaffolds suggests that ASCs embedded in natural scaffolds (*n* = 13), such as ECM, adipose tissue extract, and small intestine submucosa extract [23, 26–29, 36], differentiate adipogenically in vivo. ASCs embedded in synthetic scaffolds (*n* = 2) did not differentiate [14, 20] unless predifferentiated (*n* = 2) [25, 34]. Predifferentiating the ASCs prior to application generally resulted in increased adipogenesis in vivo regardless of the scaffold subgroup [23, 25, 27, 28, 34–36, 38]. Interestingly, one article described increased neoadipogenesis in a synthetic scaffold with noninduced ASCs. However, the scaffold did not integrate successfully with the host tissue [25]. Based on these results, it would be paramount to discover whether the synthetic scaffold could be modified, e.g., combined with a natural scaffold, to retain neoadipogenesis while improving scaffold integration.

One study found significantly increased neoadipogenesis after the addition of fat to a natural scaffold embedded with ASCs [18]. The addition of ASCs to fat resembles the latest attempted method for soft tissue augmentation. Members of our research group have previously found that enriching fat grafts with autologous, ex vivo expanded ASCs was beneficial for graft survival compared to conventional fat grafting [8]. Recently, these findings were confirmed in a randomized clinical breast augmentation trial [9]. Whether the increased neoadipogenesis results from ASC-enriched fat alone or the synergism of fat, ASCs and the scaffold was not assessed.

A study utilizing natural scaffolds (small intestine submucosa extract and adipose tissue extract) reported that ASCs alone induced lipid droplet formation but without the functions and components of normal lipid droplets [29]. In general, ASCs embedded in natural scaffolds induce neoadipogenesis and differentiation of ASCs prior to application [23, 36, 38]. Adding fat to an ASC-embedded scaffold would probably improve this effect further.

### Vascularization

Neovascularization is fundamental for new tissue formation. Eight of the 13 studies utilizing natural scaffolds reported positive results of vascularization when embedding dASCs [19, 26–29, 32, 36, 37]. However, this effect was lost when employing undifferentiated ASCs [18, 24, 30, 31], indicating that adipogenic induction might enhance vascularization in a natural scaffold.

Of the studies that utilized synthetic scaffolds, only two assessed neovascularization [25, 34]. One of which solely stated, “blood supply in the engineered tissue remains a problem” [25]. The other study successfully detected vascularization when embedding both dASCs and ASCs via histological examination and positive CD31 and vWF staining [34].

The formation of neovascularization in the combinatory scaffolds was reported in four [21, 22, 33, 37] out of five studies [21, 22, 33, 35, 37]. In contrast to the natural scaffolds, neovascularization in combinatory scaffolds only occurred with the use of undifferentiated ASCs. However, no vascularization was observed in the combinatory scaffold embedded with dASCs [35]. These results could indicate that the adipogenic induction of ASCs might not enhance vascularization in combinatory scaffolds. This conclusion agrees with the report from the study in which both dASCs and ASCs were assessed in a combinatory scaffold [38]. This study found that blood vessel development was unaffected despite the use of adipogenic induction. The adipogenic effect on neovascularization remains to be characterized, along with the observation that the effect is lost when applied to combinatory scaffolds.

The origin of neovessels was reported to be formed by host tissue in natural-/dASCs [37], synthetic-/ASCs [34], and combinatory/ASC scaffolds [33]. One study reported that dASCs induced vascularization in the host tissue, whereas ASCs induced vascularization in the donor tissue when embedded in synthetic scaffolds [34].

Two articles found no significant difference in vascularization by the end of the studies when comparing injected ASCs alone and ASCs embedded in scaffolds [22, 31]. However, one of the studies described a significantly faster initial vessel formation in the ASC-embedded hydrogel group compared to the control groups, but this difference between the groups was aligned by day 21 [31]. This limited number of studies indicates that dASCs in combination with a natural scaffold or ASCs employed with a combinatory scaffold improve neoangiogenesis.

### Wound healing

Wound healing is a complex process. In short, it can be divided into four distinct phases: the hemostasis phase, the inflammatory phase, the proliferative phase, and the remodeling phase. ASCs are known for their regenerative properties; they secrete VEGF, fibroblast-like growth factor, platelet-derived growth factor promoting angiogenesis [41], which provides the newly formed tissue with oxygen and nutrition faster [42]. Furthermore, ASCs promote ECM reconstruction by regulating the ratio of collagen type III:type I, transforming growth factor-β3:transforming growth factor-β1 and matrix metalloproteinases-3:matrix metalloproteinase-1. These shifts in ratios decrease fibrosis, which contributes to scar remodeling [43]. Knowing these distinct phases and ASC functions, the application of ASCs is evident in wound healing.

The five included studies assessing wound healing utilized undifferentiated ASCs injected dermally in natural or combinatory scaffolds [21, 22, 30–32]. All but one study [21] reported significantly faster (*P* < 0.05) wound healing, adherent to the theoretical advantage of adding ASCs to wounds. The latter study was leveled out by contracture of the control wounds. Although no difference was found regarding healing time, the wounds treated with scaffolded ASCs trended towards better re-epithelization and increased vascularization [21].

Increased epithelial thickness was reported to be improved in two natural scaffolds embedded in ASCs [30, 31]. Faster re-epithelialization was reported in two studies, one natural [31] and one combinatory [22]. Three of the five papers reported increased vascularization in natural [32] and combinatory [21, 22] scaffolds compared with their controls. Even with this limited number of studies on wound healing, these results indicate that ASC-embedded scaffolds improve wound quality. This proves to be a great prospect for the treatment of chronic wounds; however, chronic disease models were excluded from this review, and these scaffolds have been reported to accelerate diabetic wound healing and enhance ASC cytokine secretion [44].

## Discussion

Although statistical comparison was limited by clinical heterogeneity, the included studies have provided us with important knowledge within the advancing field of ASC treatments for soft tissue restoration and wound healing.

With respect to the investigated outcomes, four natural, two synthetic, and two combinatory scaffolds embedding ASCs significantly increased outcome measurements compared to ASCs alone [14, 21, 22, 25, 27, 29, 32]. Three scaffolds did not reach statistical significance but trended towards improved treatment embedded in a natural scaffold [19, 32]. It seems that scaffolds generally enhance ASC-associated outcomes. This may be attributed to the mechanical protection provided by the scaffold, leading to sustained cell viability in vivo.

Another important consideration in designing scaffolds for ASC treatment, uncovered in this review, is whether to use natural, synthetic, or combinatory materials. A natural scaffold is recommended for fat generation due to the uncovered adipogenically inducing effects of undifferentiated ASCs in combination with natural materials. However, entirely natural scaffolds generally have reduced mechanical stability, and as discovered in this review, the mechanical strength and protective qualities of the scaffold are of great importance for cell viability and differentiation. At present there is no data evaluating the functional differences between natural derived scaffolds embedding ASCs. However, the native biological function must be expected by natural scaffolds, if they are injected or implanted in the same environmental niche as harvested.

An important aspect to consider before the clinical application of scaffolds is manageability. This needs to be addressed when selecting a suitable scaffold for ASC transplantation. This subject is highly overlooked or only superficially considered in most studies. Questions such as the following: how well do the cells mix with the scaffold solution, what is the required time for embedding a clinical dose, will the scaffold be able to support a clinically relevant dose, and is the embedding procedure to be done in a closed system manner in the laboratory or in the OR? Last, will the scaffold be approved for clinical use in humans?

The common use of immunodeficient animal models in this review is a potential source of inaccuracy when translating these strategies to clinical applications in humans. Furthermore, human and murine MSCs differ in their immunomodulatory mechanisms and cannot be directly compared with each other [45]. The use of human ASCs in animal models poses another interspecies problem regarding immune responses. By injecting human ASCs into an immunodeficient animal, the donor cells and recipient tissue will not respond adequately to one another, as they would after autologous or allogenic transplantation in an immunocompetent human. The immunomodulatory and anti-inflammatory factors mediated by the injected cells may therefore impair outcomes. A solution to this problem is emerging with the use of humanized murine models, in which parts of the human immune system are incorporated into mice [46].

### Potential solutions to current obstacles in the field of ASC treatments

As mentioned, the protective qualities and therefore the mechanical strength of a scaffold largely influence cell viability. However, the stiffness of scaffolds is limited by the needle gauge. In response to this hindrance, some authors successfully designed thermoresponsive scaffolds. In short, these scaffolds were designed to be viscous and protect the cells during injection, similar to every other scaffold, but to stiffen at a higher temperature [14, 31, 32]. If this approach is applied properly, ASCs could have the best possible protection during injection and gain additional mechanical protection as the scaffold stiffens postinjection. Furthermore, the thermoresponsive scaffold can be moldable, which is a major advantage for dermal injections to treat and minimize skin irregularities. Therefore, a combinatory or synthetic scaffold with thermoresponsive abilities in combination with dASCs could be favorable for achieving both increased adipogenesis, vascularization, and mechanical protection during delivery and after implantation. If utilizing a combinatory scaffold, it should be taken into consideration that vascularization could be enhanced by the use of undifferentiated ASCs.

## Conclusions

The use of scaffolds as a vehicle for ASC delivery generally improved cell viability, angiogenesis and wound healing in vivo compared to utilizing ASCs alone*.* ASCs embedded in natural materials induced more adipogenesis than ASCs embedded in synthetic materials. dASCs further increased this effect. The included studies indicate that the seeded scaffold material influences the differentiation of ASCs in vivo*.* All studies investigating the mechanical strength of ASC scaffolds reported improved outcome measurements with improved mechanical strength. The results suggest that scaffolds, in general, are favorable for ASC delivery. We recommend initiating clinical studies using scaffolds based on mechanical properties and tunability to improve ASC viability. For fat regeneration, natural scaffolds are recommended.

## Data Availability

The data that support the findings of this study are available on request from the corresponding author.

## Abbreviations

ASCs: Adipose-derived stromal cells
dASCs: Adipogenic differentiated adipose-derived stromal cells
ECM: Extracellular matrix
MSCs: Mesenchymal stem cells
PEG: Polyethylene glycol
PLGA: Poly(lactide-co-glycolide)

## References

[1] Patrikoski M, Mannerström B, Miettinen S (2019). Perspectives for clinical translation of adipose stromal/stem cells. Stem Cells Int.

[2] Atala A, Lanza R (2013). Handbook of stem cells.

[3] Kong HJ, Smith MK, Mooney DJ (2003). Designing alginate hydrogels to maintain viability of immobilized cells. Biomaterials..

[4] Aguado BA, Mulyasasmita W, Su J, Lampe KJ, Heilshorn SC (2012). Improving viability of stem cells during syringe needle flow through the design of hydrogel cell carriers. Tissue Eng A.

[5] Delay E, Garson S, Tousson G, Sinna R (2009). Fat injection to the breast: technique, results, and indications based on 880 procedures over 10 years. Aesthet Surg J.

[6] Khouri R, Del Vecchio D (2009). Breast reconstruction and augmentation using pre-expansion and autologous fat transplantation. Clin Plast Surg.

[7] Nishimura T, Hashimoto H, Nakanishi I, Furukawa M (2000). Microvascular angiogenesis and apoptosis in the survival of free fat grafts. Laryngoscope..

[8] Kølle SF, Fischer-Nielsen A, Mathiasen AB, Elberg JJ, Oliveri RS, Glovinski PV (2013). Enrichment of autologous fat grafts with *ex-vivo* expanded adipose tissue-derived stem cells for graft survival: a randomised placebo-controlled trial. Lancet..

[9] Kølle S-FT, Duscher D, Taudorf M, Fischer-Nielsen A, Svalgaard JD, Munthe-Fog L, et al. *Ex vivo*-expanded autologous adipose tissue-derived stromal cells ensure enhanced fat graft retention in breast augmentation: a randomized controlled clinical trial. Stem Cells Transl Med. 2020:1–10. 10.1002/sctm.20-0081.1-10. 10.1002/sctm.20-0081 PMC7581442 32639099

[10] Rehman J, Traktuev D, Li J (2004). Secretion of angiogenic and antiapoptotic factors by human adipose stromal cells. Circulation..

[11] Honnegowda TM, Kumar P, Udupa EGP (2015). Role of angiogenesis and angiogenic factors in acute and chronic wound healing. Plast Aesthet Res.

[12] van Dongen JA, Harmsen MC, van der Lei B, Stevens HP (2018). Augmentation of dermal wound healing by adipose tissue-derived stromal cells (ASC). Bioengineering (Basel).

[13] Youngblood RL, Truong NF, Segura T, Shea LD (2018). It’s all in the delivery: designing hydrogels for cell and non-viral gene therapies. Mol Ther.

[14] Cai L, Dewi RE, Heilshorn SC (2015). Injectable hydrogels with in situ double network formation enhance retention of transplanted stem cells. Adv Funct Mater.

[15] Dhandayuthapani B, Yoshida Y, Maekawa T, Kumar S (2011). Polymeric scaffolds in tissue engineering application: a review. Int J Polym Sci.

[16] Li Y, Rodrigues J, Tomás H (2012). Injectable and biodegradable hydrogels: gelation, biodegradation and biomedical applications. ChemSoc Rev.

[17] Xie J, Zhang D, Zhou C (2018). Substrate elasticity regulate adipose-derived stromal cell differentiation towards osteogenesis and adipogenesis through β-catenin transduction. Act Bio.

[18] Chen YS, Hsueh YS, Chen YY, Lo CY, Tai HC, Lin FH (2017). Evaluation of a laminin-alginate biomaterial, adipocytes, and adipocyte-derived stem cells interaction in animal autologous fat grafting model using 7-Tesla magnetic resonance imaging. J Mater Sci Mater Med.

[19] Sumi Y, Ishihara M, Kishimoto S, Takikawa M, Doumoto T, Azuma R (2013). Transplantation of inbred adipose-derived stromal cells in rats with plasma gel containing fragmin/protamine microparticles and FGF-2. J Biomed Mater Res B Appl Biomater.

[20] Derby BM, Dai H, Reichensperger J, Cox L, Harrison C, Cosenza N (2014). Adipose-derived stem cell to epithelial stem cell transdifferentiation: a mechanism to potentially improve understanding of fat grafting's impact on skin rejuvenation. Aesthet Surg J.

[21] Dong Y, Hassan WU, Kennedy R, Greiser U, Pandit A, Garcia Y (2014). Performance of an in situ formed bioactive hydrogel dressing from a PEG-based hyperbranched multifunctional copolymer. Acta Biomater.

[22] Dong Y, Sigen A, Rodrigues M, Li X, Kwon SH, Kosaric N, et al. Injectable and tunable gelatin hydrogels enhance stem cell retention and improve cutaneous wound healing. Adv Funct Mater. 2017;27:1606619.

[23] Jing W, Lin Y, Wu L, Li X, Nie X, Liu L (2007). Ectopic adipogenesis of preconditioned adipose-derived stromal cells in an alginate system. Cell Tissue Res.

[24] Linh NT, Abueva CD, Lee BT (2017). Enzymatic in situ formed hydrogel from gelatin-tyramine and chitosan-4-hydroxylphenyl acetamide for the co-delivery of human adipose-derived stem cells and platelet-derived growth factor towards vascularization. Biomed Mater.

[25] Choi YS, Cha SM, Lee YY, Kwon SW, Park CJ, Kim M (2006). Adipogenic differentiation of adipose tissue derived adult stem cells in nude mouse. Biochem Biophys Res Commun.

[26] Choi JS, Yang HJ, Kim BS, Kim JD, Kim JY, Yoo B (2009). Human extracellular matrix (ECM) powders for injectable cell delivery and adipose tissue engineering. J Control Release.

[27] Kim WS, Mooney DJ, Arany PR, Lee K, Huebsch N, Kim J (2012). Adipose tissue engineering using injectable, oxidized alginate hydrogels. Tissue Eng A.

[28] Cheung HK, Han TT, Marecak DM, Watkins JF, Amsden BG, Flynn LE (2014). Composite hydrogel scaffolds incorporating decellularized adipose tissue for soft tissue engineering with adipose-derived stem cells. Biomaterials..

[29] Wang JQ, Fan J, Gao JH, Zhang C, Bai SL (2013). Comparison of *in vivo* adipogenic capabilities of two different extracellular matrix microparticle scaffolds. Plast Reconstr Surg.

[30] Machula H, Ensley B, Kellar R (2014). Electrospun tropoelastin for delivery of therapeutic adipose-derived stem cells to full-thickness dermal wounds. Adv Wound Care (New Rochelle).

[31] Kim EJ, Choi JS, Kim JS, Choi YC, Cho YW (2016). Injectable and thermosensitive soluble extracellular matrix and methylcellulose hydrogels for stem cell delivery in skin wounds. Biomacromolecules..

[32] Cheng NC, Lin WJ, Ling TY, Young TH (2017). Sustained release of adipose-derived stem cells by thermosensitive chitosan/gelatin hydrogel for therapeutic angiogenesis. Acta Biomater.

[33] Wu J, Zhang K, Yu X, Ding J, Cui L, Yin J (2017). Hydration of hydrogels regulates vascularization *in vivo*. Biomater Sci.

[34] Zhang K, Song L, Wang J, Yan S, Li G, Cui L (2017). Strategy for constructing vascularized adipose units in poly(l-glutamic acid) hydrogel porous scaffold through inducing *in-situ* formation of ASCs spheroids. Acta Biomater.

[35] Cho SW, Kim SS, Rhie JW, Cho HM, Choi CY, Kim BS (2005). Engineering of volume-stable adipose tissues. Biomaterials..

[36] Hong KY, Yim S, Kim HJ, Jin US, Lim S, Eo S (2018). The fate of the adipose-derived stromal cells during angiogenesis and adipogenesis after cell-assisted lipotransfer. Plast Reconstr Surg.

[37] Dhillon J, Young SA, Sherman SE, Bell GI, Amsden BG, Hess DA (2019). Peptide-modified methacrylated glycol chitosan hydrogels as a cell-viability supporting pro-angiogenic cell delivery platform for human adipose-derived stem/stromal cells. J Biomed Mater Res A.

[38] Storck K, Fischer R, Buchberger M, Haller B, Regn S (2017). Delivered adipose-derived stromal cells improve host-derived adipose tissue formation in composite constructs *in vivo*. Laryngoscope..

[39] Chan BP, Leong KW (2008). Scaffolding in tissue engineering: general approaches and tissue-specific considerations. Eur Spine J.

[40] Rasmussen BS, Sørensen CL, Kurbegovic S, Ørholt M, Talman MM, Herly M (2019). Cell-enriched fat grafting improves graft retention in a porcine model: a dose-response study of adipose-derived stem cells versus stromal vascular fraction. Plast Reconstr Surg.

[41] Navneet D, Viraj M, Rajni D, Yue-Hua D, Feng-Chou T, Wing-Ping D (2018). Revisiting the advances in isolation, characterization and secretome of adipose-derived stromal/stem cells. Int J Mol Sci.

[42] Lombardi F, Palumbo P, Augello FR, Cifone MG, Cinque B, Giuliani M (2019). Secretome of adipose tissue-derived stem cells (ASCs) as a novel trend in chronic non-healing wounds: an overview of experimental *in vitro* and *in vivo* studies and methodological variables. Int J Mol Sci.

[43] Wang L, Hu L, Zhou X, Xiong Z, Zhang C, Shehada HMA (2017). Exosomes secreted by human adipose mesenchymal stem cells promote scarless cutaneous repair by regulating extracellular matrix remodelling. Sci Rep.

[44] Hopfner U, Aitzetmueller MM, Neßbach P, Hu MS, Machens HG, Maan ZN, et al. Fibrin glue enhances adipose-derived stromal cell cytokine secretion and survival conferring accelerated diabetic wound healing. Stem Cells Int. 2018;2018:1353085. 10.1155/2018/1353085 PMC6313983 30662467

[45] Su J, Chen X, Huang Y, Li W, Li J, Cao K (2014). Phylogenetic distinction of iNOS and IDO function in mesenchymal stem cell-mediated immunosuppression in mammalian species. Cell Death Differ.

[46] Mehler VJ, Burns C, Moore ML (2019). Concise review: exploring immunomodulatory features of mesenchymal stromal cells in humanized mouse models. Stem Cells.

